# Infective Endocarditis With Negative Cardiac Multimodal Imaging: A Case Report

**DOI:** 10.7759/cureus.105521

**Published:** 2026-03-19

**Authors:** Rei Chin, Balakrishna Kumar

**Affiliations:** 1 Stroke Medicine, Portsmouth Hospitals University NHS Trust, Portsmouth, GBR

**Keywords:** 18f-fluorodeoxyglucose positron emission tomography (18f-fdg pet), echocardiography, endocarditis, multimodal imaging, multi-territory stroke

## Abstract

Multimodal imaging is a key part of infective endocarditis (IE) diagnostic guidelines. However, we present a case of confirmed IE where evidence of disease was absent on multiple imaging modalities.

A patient was admitted following multi-territory ischemic strokes. He had had an aortic root graft with tissue aortic valve replacement and a previous ischemic stroke. During admission, the patient developed pyrexia of unknown origin. Four blood cultures were positive for *Staphylococcus haemolyticus.* Transthoracic and transoesophageal echocardiogram, as well as ^18^F-FDG positron emission tomography/computed tomography (FDG PET/CT) failed to show any signs of IE. However, there was sufficient evidence to diagnose *definite endocarditis* according to the Modified Duke Criteria.

This case report highlights the limitations of imaging in IE diagnosis. Further research into the sensitivity of FDG PET/CT as a diagnostic tool for IE needs to be conducted. This is important to avoid underdiagnosis of IE due to overreliance on radiological evidence.

## Introduction

Infective endocarditis (IE) is a disease with major global impact due to its high morbidity and mortality [1,2]. It is commonly diagnosed with the Modified Duke Criteria. At least one major criterion must be met to diagnose definite IE. Radiological evidence has traditionally been one of two major criteria and therefore plays a large role in the diagnostic algorithm for IE [3]. Only in 2023 did the International Society for Cardiovascular Infectious Diseases propose the addition of a third major criterion - surgical evidence [4]. The minor criteria are clinical and biochemical manifestations of IE.

Increasingly, there has been evidence supporting the use of ^18^F-FDG positron emission tomography/computed tomography (FDG PET/CT) in the diagnosis of IE [5]. It has been added as an accepted imaging modality into the Modified Duke Criteria as well as the ESC Guidelines for the management of endocarditis. The pooled sensitivity and specificity for all types of IE are high [6], but the sensitivity is far higher for prosthetic valve disease compared to native valve disease.

Since 2015, the ESC Guidelines for the management of IE have advocated for the use of multimodal imaging in the diagnosis of IE [7]. The rationale is to improve sensitivity in the detection of IE. These imaging modalities include cardiac CT as well as white blood cell single photon emission computed tomography/CT (WBC SPECT/CT), in addition to echocardiography and FDG PET/CT.

We present a case where radiological evidence of IE was absent on transthoracic echocardiogram (TTE), transesophageal echocardiogram (TEE), and FDG PET/CT. The diagnosis of IE was confirmed using the modified Duke Criteria, with only major microbiological evidence and three minor criteria. This case report, therefore, highlights the danger of over-reliance on imaging in IE diagnosis. Missed diagnosis of IE is serious because severe complications and death can result from untreated IE.

## Case presentation

A man in his sixties was admitted with new-onset confusion. The patient had undergone an aortic root graft with tissue aortic valve replacement 10 years earlier for aortic root dilatation and a bicuspid aortic valve, and a valve-in-valve transcatheter aortic valve implantation three years earlier for severe aortic regurgitation. He had also been treated with lifelong apixaban for thrombi found on the aortic valve replacement leaflets two years earlier.

**Figure 1:**
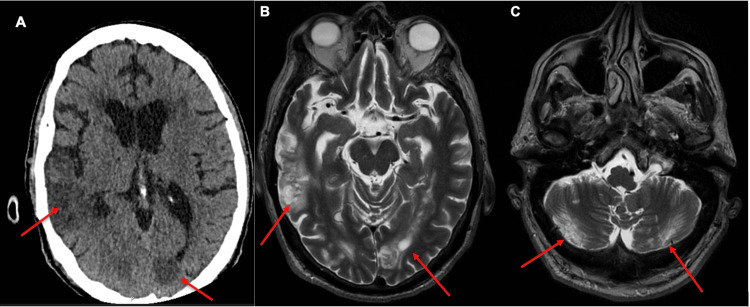
(A) Computed tomography showing bilateral infarcts involving both the anterior and posterior vascular territories (arrows); (B, C) T2 Periodically Rotated Overlapping Parallel Lines with Enhanced Reconstruction (PROPELLER) images showing bilateral cerebral and cerebellar infarcts (arrows), respectively.

CT and subsequently magnetic resonance imaging (MRI) were performed (Figure 1). These showed multiple acute bilateral ischemic strokes affecting both the anterior and posterior circulation territories, as well as the cerebral and cerebellar regions.

Given the history of recurrent, bilateral ischemic strokes, the patient was investigated for underlying causes. Initial review of the CT angiogram suggested possible signs of vasculitis, but two neuroradiologists at tertiary centers contested this. A magnetic resonance angiogram was subsequently performed and did not show evidence of vasculitis. The patient was too confused to tolerate an MRI with gadolinium contrast. The patient had a fluctuating level of cognition and attention, but there were no obvious signs of meningitis. Lumbar puncture, infectious screen, and systemic vasculitis screen did not show any abnormalities. The electroencephalogram did not show signs of generalized encephalopathy consistent with central nervous system vasculitis. Rheumatoid factor and erythrocyte sedimentation rate were elevated, whereas anti-cyclic citrullinated peptide antibodies were negative. Blood counts were not suggestive of lymphoma, and CT scans did not show lymphoma. The antiphospholipid screen was also negative. The myeloma screen was negative.

**Figure 2:**
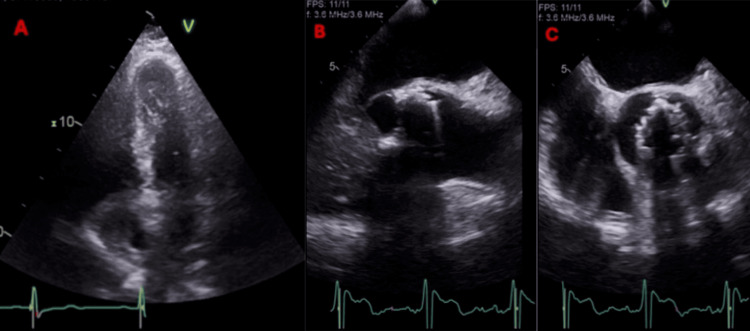
Echocardiographic images showing no evidence of infective endocarditis. (A) Four-chamber view on transthoracic echocardiography (TTE). (B) Long-axis view on transesophageal echocardiography (TEE). (C) Mid-esophageal short-axis view on TEE. No vegetations were visualized on any valves. Panel A shows the mitral and tricuspid valves. Panel B shows the left ventricular outflow tract with the aortic valve replacement in situ. Panel C shows the aortic valve replacement.

**Figure 3:**
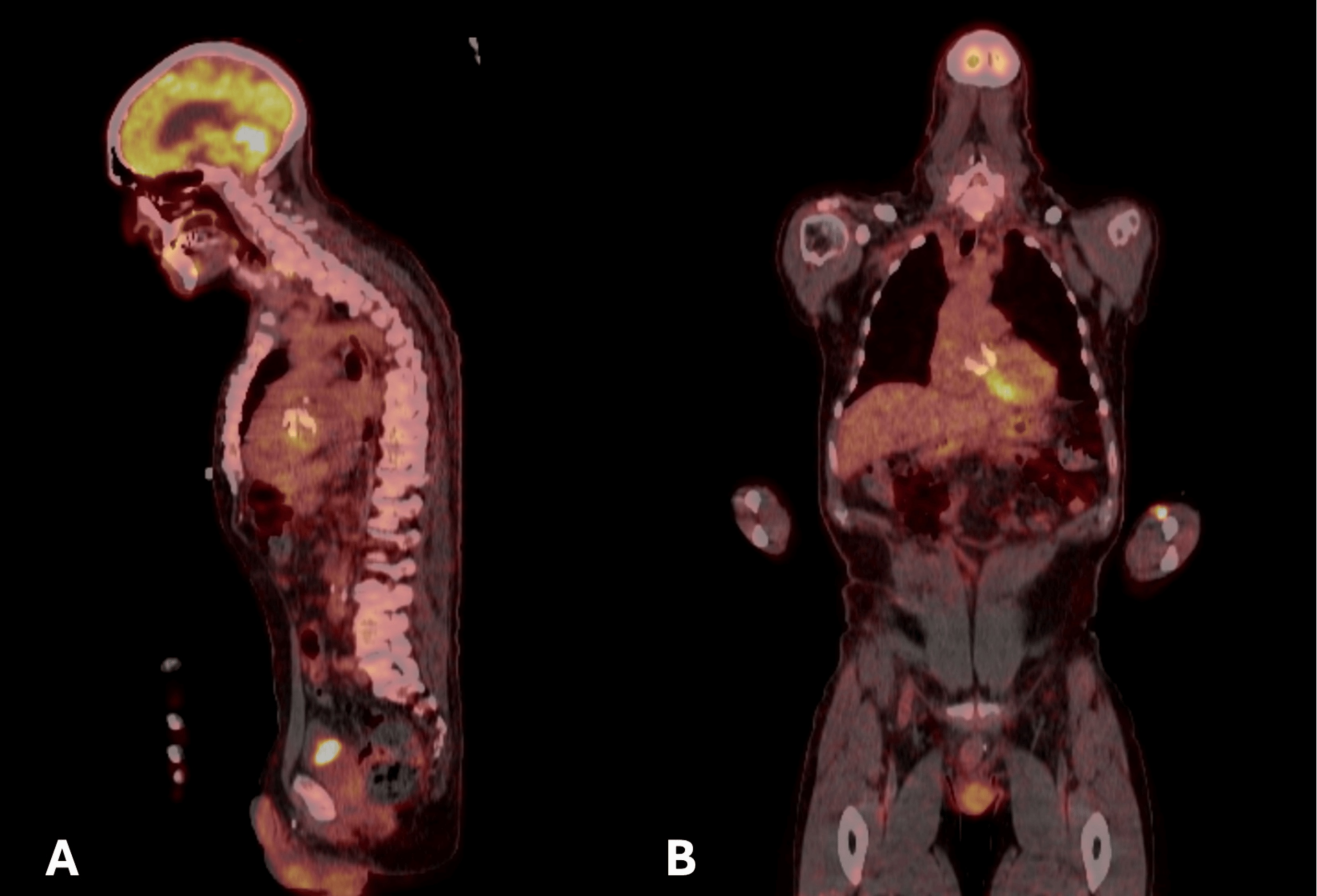
18F-FDG positron emission tomography/computed tomography images showing no evidence of infective endocarditis: (A) sagittal plane; (B) coronal plane. There was no tracer accumulation at the aortic valve replacement to suggest an underlying infective process. Left ventricular myocardial uptake was reported as physiological.

In weeks 2 and 5 of admission, the patient had recurrent fevers above 38 °C. Respiratory viral swabs and urine cultures remained negative throughout this period. A CT scan of the thorax, abdomen, and pelvis showed no foci of deep-seated infection or widespread infarcts suggestive of IE. There was also no evidence of IE on TTE and TEE (Figure 2). An FDG PET/CT scan was performed in week three of admission (Figure 3), which did not show a clear source of infection. No chronic infectious or autoimmune cause of the pyrexia of unknown origin could be identified. Within the first month of admission, four blood cultures were performed by numerous clinicians, all of which cultured positive for *Staphylococcus haemolyticus*. Given the repeated positive blood cultures, this was deemed not to be a contaminant. Table 1 shows a comprehensive list of laboratory investigation results. On examination, a pre-existing murmur was noted, and finger clubbing was present.

**Table 1:** Comprehensive laboratory findings. *Abnormal laboratory values. CMV, cytomegalovirus; IgG, immunoglobulin G; IgM, immunoglobulin M; HIV, human immunodeficiency virus; VZV, varicella zoster virus; IE, infective endocarditis; ESR, erythrocyte sedimentation rate; C3, complement component 3; C4, complement component 4; cANCA, cytoplasmic anti-neutrophil cytoplasmic antibody; pANCA, perinuclear anti-neutrophil cytoplasmic antibody; anti-CCP antibody, anti-cyclic citrullinated peptide antibody; β2GP1, anti-β2-glycoprotein 1 antibodies; CSF, cerebrospinal fluid; HSV, herpes simplex virus

Parameter	Patient value	Reference range
Blood microscopy and culture	Mixed coagulase-negative *Staphylococcus* on one occasion; *Staphylococcus haemolyticus *on four occasions	N/A
Respiratory viral swab	Influenza, respiratory syncytial virus, and SARS-CoV-2 were not detected during two separate instances of fever.	N/A
Urine microscopy	No evidence of infection during two separate instances of fever	N/A
T-SPOT TB assay	Negative	N/A
Syphilis antibody	Negative	N/A
CMV IgG and IgM	Not detected	N/A
EBV capsid IgG and IgM, and nuclear antigen antibodies	Not detected	N/A
HIV antigen/antibody combination test	Not detected	N/A
Hepatitis B surface antigen antibody	Not detected	N/A
Hepatitis C antibody	Not detected	N/A
VZV IgG	Detected	N/A
Procalcitonin	0.07-0.13 μg/L before antibiotics started for IE	N/A
ESR	114-125 mm/hour*	2-14 mm/hour
Rheumatoid factor	605 IU/mL*	0-14 IU/mL
Anti-nuclear antibodies	Negative	N/A
C3	1.46 g/L	0.90-1.80 g/L
C4	0.18 g/L	0.10-0.40 g/L
cANCA	Negative	N/A
pANCA	Negative	N/A
Anti-CCP antibody	<2 RU/mL	0-20 RU/mL
Anti-cardiolipin IgG	<8 GPL U/mL	0-9 GPL U/mL
Anti-cardiolipin IgM	10-11 MPL U/mL	0-7 MPL U/mL
Immunoglobulin profile	Reactive profile not indicative of myeloma	N/A
β2GP1	<1.40 U/mL	0.0-20.0 U/mL
Urine protein creatinine ratio	22 mg/mmol	1-23 mg/mmol
CSF microscopy	Organisms not seen; white blood cells <2 per μL; red blood cells <2 per μL.	N/A
CSF culture	No growth after 48 hours of incubation	N/A
CSF nucleic acid detection	VZV, HSV, and enterovirus were not detected	N/A
CSF VZV IgG	Not detected	N/A
CSF total protein	0.82-0.91 g/L	0.15-0.45 g/L
CSF glucose	3.2 mmol/L	2.2-3.9 mmol/L
Paired serum and CSF oligoclonal banding	Oligoclonal bands were present in both serum and CSF, with identical banding patterns, suggesting a systemic inflammatory process.	N/A

Using the Modified Duke Criteria [4], definite IE was diagnosed (Table 2) based on one major criterion and more than three minor criteria. The presentation of cardioembolic ischemic strokes would be in keeping with a left heart prosthetic valve. IE antibiotic treatment was started on day 35 of admission based on the microbiologist’s guidance. Intravenous vancomycin and gentamicin were commenced and later changed to ceftriaxone; these were administered alongside oral rifampicin. The patient’s mental health deteriorated as his admission prolonged, and his cognition remained poor secondary to the ischemic strokes. It was therefore difficult to treat the IE with intravenous antibiotics, which required regular venipuncture for therapeutic monitoring. Nevertheless, the antibiotic course was completed on day 69 of admission, and the patient was discharged two days later to a residential home due to persistent dementia following the stroke.

**Table 2:** Basis of definite infective endocarditis diagnosis using the Modified Duke Criteria. Definite infective endocarditis diagnosed - 1 major criterion + ≥3 minor criteria.

Criteria		Evidence
Major	Microbiologic	1. i *Staphylococcus haemolyticus* grown in multiple blood cultures - a typical microorganism causing infective endocarditis (coagulase-negative *Staphylococcus*).
Minor	Predisposition	History of prosthetic heart valve
	Fever	Documented fever above 38.0 °C
	Vascular phenomena	Clinical and radiological evidence of arterial emboli - ischemic stroke
	Immunologic phenomena	Positive rheumatoid factor

## Discussion

IE can present in many ways, and symptoms are often subtle or non-specific. This case report highlights the importance of a holistic approach to the diagnosis of IE. As evidence is increasing that imaging modalities such as FDG PET/CT are reliable in aiding the identification of IE [6], there is a risk of overreliance on radiologic evidence as the basis for diagnosis. Consequently, the incidence of IE may be underestimated. Undiagnosed IE results in severe morbidity, therefore contributing to significant financial and emotional impact on society and its healthcare system. To prevent this, clinicians must not neglect the physical examination and biochemical investigations, especially when multimodal imaging fails to show evidence of IE. 

Sensitivity of FDG PET/CT for IE identification is much higher for prosthetic valves at 0.86 compared to that of native valves at 0.31 [6]. Given that the case presented was of prosthetic valve endocarditis, the inability to identify IE on FDG PET/CT is surprising. Some evidence suggests that false-negative results can occur in patients with long-term antibiotic use [8]. However, the only antibiotic administered before the FDG PET/CT was a seven-day course of co-amoxiclav, and *S. haemolyticus *is known to have high resistance to penicillins [9]. This is therefore unlikely to explain the negative FDG PET/CT. Another possible explanation is that the affected area is a moving vegetation, meaning that the moving focus of high FDG uptake is not captured on the scan [10]. However, given that there were no vegetations seen on echocardiogram, the more likely explanation is that the area of IE was small with low metabolic activity. An extensive literature search was performed: previous reports of TTE-, TEE-, and FDG PET/CT-negative IE are rare.

Some diagnostic algorithms suggest the use of WBC SPECT/CT if FDG PET/CT is negative for IE, but clinical suspicion remains [11]. This was not performed in the case presented. However, evidence suggests that the sensitivity of WBC SPECT/CT is less than that of FDG PET/CT in the detection of IE [12].

Central to the ESC diagnostic guidelines is multimodal imaging - the combination of cardiac CT, echocardiogram, and FDG PET/CT [1]. However, there is currently limited evidence on the sensitivity and specificity of multimodal imaging in IE diagnosis [13]; further research is required in this area. Additionally, research shows an improvement in sensitivity and specificity of FDG PET/CT over time [6]. It is therefore necessary to continue data collection as this imaging modality matures and the sample size for research increases.

## Conclusions

We presented a case of prosthetic valve IE where the diagnosis was made despite negative multimodal imaging. Other evidence of IE, such as clinical and microbiological criteria were used instead to make the diagnosis. Clinicians must remember the limitations of radiological methods while investigating suspected IE in order to avoid underdiagnosis. Clinical evidence and laboratory findings are equally important in IE diagnosis. Further research is required to better characterize the role of newer imaging techniques, such as FDG PET/CT, in the diagnostic algorithm of IE.
